# The intestinal γδ T cells: functions in the gut and in the distant organs

**DOI:** 10.3389/fimmu.2023.1206299

**Published:** 2023-06-16

**Authors:** Guo-Qing Li, Jiliang Xia, Weihong Zeng, Weijia Luo, Logen Liu, Xi Zeng, Deliang Cao

**Affiliations:** ^1^ Department of Gastroenterology, Clinical Research Center, the Second Affiliated Hospital, Hengyang Medical School, University of South China, Hengyang, Hunan, China; ^2^ Hunan Provincial Key Laboratory of Basic and Clinical Pharmacological Research on Gastrointestinal Tumors, The Second Affiliated Hospital, Hengyang Medical School, University of South China, Hengyang, China; ^3^ Hunan Province Key Laboratory of Cancer Cellular and Molecular Pathology, Cancer Research Institute, Hengyang Medical School, University of South China, Hengyang, Hunan, China; ^4^ Department of Oncology, The First Affiliated Hospital, Hengyang Medical School, University of South China, Hengyang, Hunan, China

**Keywords:** mucosal immunity, intestinal γδ T cells, lymphopoiesis, γδ T−epithelial remodeling, and gut γδ T-brain injury repair

## Abstract

Located in the frontline against the largest population of microbiota, the intestinal mucosa of mammals has evolved to become an effective immune system. γδ T cells, a unique T cell subpopulation, are rare in circulation blood and lymphoid tissues, but rich in the intestinal mucosa, particularly in the epithelium. *Via* rapid production of cytokines and growth factors, intestinal γδ T cells are key contributors to epithelial homeostasis and immune surveillance of infection. Intriguingly, recent studies have revealed that the intestinal γδ T cells may play novel exciting functions ranging from epithelial plasticity and remodeling in response to carbohydrate diets to the recovery of ischemic stroke. In this review article, we update regulatory molecules newly defined in lymphopoiesis of the intestinal γδ T cells and their novel functions locally in the intestinal mucosa, such as epithelial remodeling, and distantly in pathological setting, e.g., ischemic brain injury repair, psychosocial stress responses, and fracture repair. The challenges and potential revenues in intestinal γδ T cell studies are discussed.

## 1 Introduction

The gut mucosa composed of the epithelium, basement membrane, and lamina propria separates a milieu enriched with various microbes and food antigens from the submucosa clear of any external pathogens (1). The intestinal epithelium consists of a single layer of intestinal epithelial cells (IECs), which represents the largest epithelial barrier of adult mammals, up to 200 ~ 400 square meters in humans (2); gut commensals are the largest microbiota on earth, up to 10^12^ microbes/gram contents in the colon (3). Therefore, the gut mucosa faces a constant threat of luminal pathogens, and the intestinal epithelium is an important barrier for the prevention of harmful substance invasion and a vital regulator of intestinal immunity (4).

**Figure 1:**
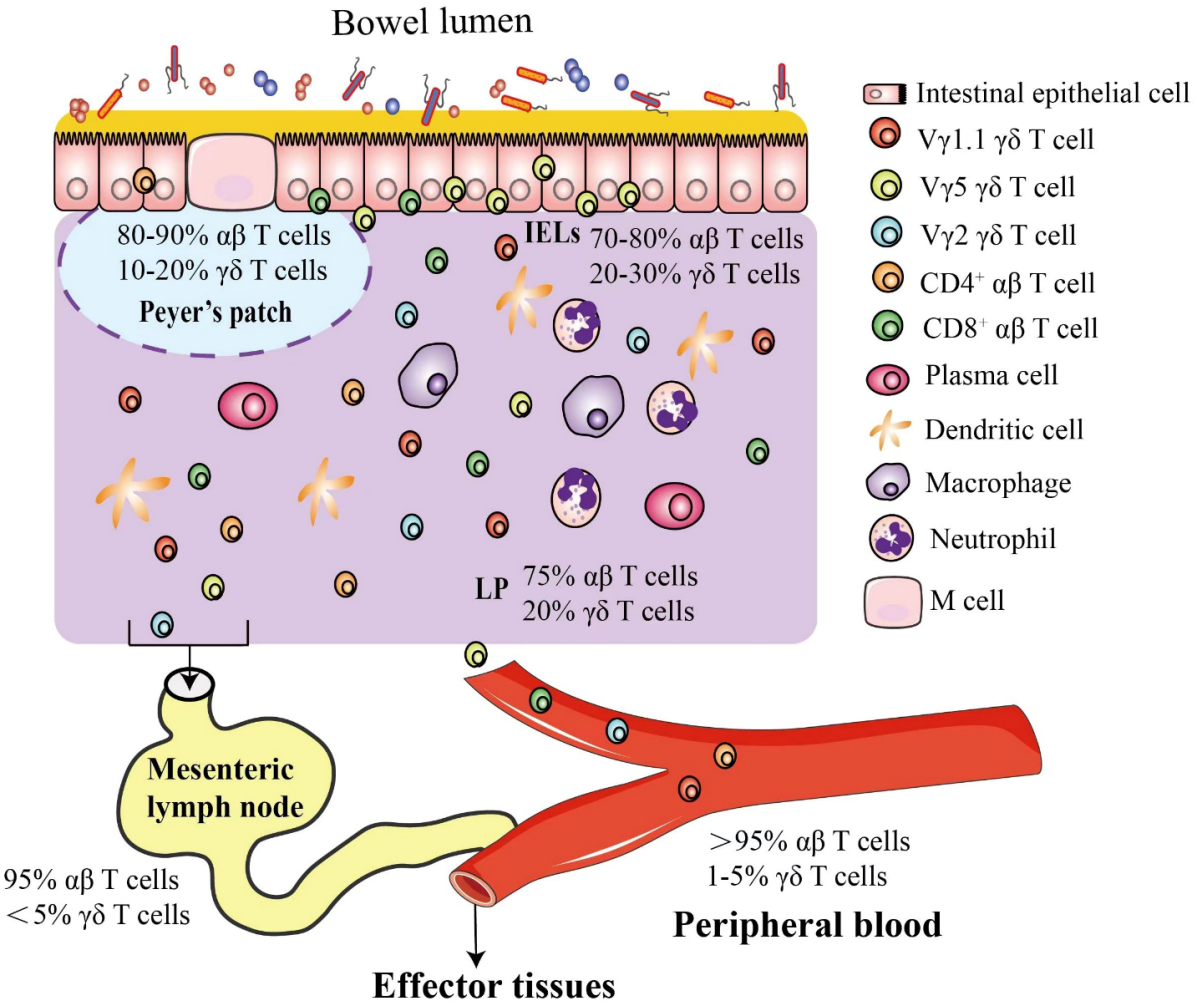
Mouse intestinal γδ T cells. The intestinal epithelium is composed of a single layer of cells that separate microbes in the lumen from the lamina propria. In response to high risk of pathogen invasion, the most complex immune system evolves in the intestine, including γδ and αβ intraepithelial lymphocytes interspersed throughout the epithelium and a plethora of immune cells in the lamina propria, such as γδ T cells, αβ T cells, dendritic cells, macrophages and neutrophils. In mice, the γδ T cells are classified by different Vγ chains, and the Vγ5^+^ γδ T cells are most popular intraepithelial γδ T cells in the intestinal epithelium. Most T cells in peripheral blood (PB) are αβ T cells, and γδ T cells account for about 1-5% (55~120 γδ T cells/µL), but in the intestinal mucosa, specific subsets of γδ T cells are enriched as shown.

The intestine has the most complex immune system and the largest repertoire of immune cells in the body, which consists of mesenteric lymph nodes (MLNs), Peyer’s patches (PPs), diffuse lymphoid structures (e.g., cryptopatches in lamina propria) and immune cells (**Figure 1**) (5, 6). T cells are scattered in lamina propria, named lamina propria lymphocytes, or reside between epithelial cells, referred to as intraepithelial lymphocytes (IELs) (7, 8). T cells are divided into αβ T and γδ T cells based on heterodimer surface receptors, i.e., T-cell receptor (TCR). TCR of αβ T cells consists of one α chain and one β chain while γδ T cells contain a γ chain and a δ chain. Intestinal intraepithelial T cells include αβ T and γδ T cells (known as γδIELs) (9, 10). In mouse intestinal epithelium, Vγ5^+^ γδ T (Garman’s System (11)) cells are most abundant (12), but Vδ1 Vγ2^+^ γδ T cells are rich in the human epithelium (13, 14).

Different from the classic and well-known CD4^+^ helper and CD8^+^ cytotoxic αβ T cells, γδ T cells are unique and MHC-unrestricted (15, 16). They have a wide range of functional plasticity through a variety of mechanisms, including the production of cytokines (e.g., IFN-γ, TNF-α, and IL-17) and chemokines (e.g., IP-10 and lymphokines), release of perforin and granzymes, and interaction with epithelial cells, monocytes, dendritic cells (DC), neutrophils, and B cells as well (15, 17–20). Therefore, γδ T cells can function as innate immune cells to serve as the first line of intestinal defense, but also shape early adaptive immune responses in anti-infection immunity (21). In the intestine, γδ T cells are the critical component of mucosal immunity, regulating epithelial homeostasis and immune response and participating in various physiological and pathological processes, such as inflammatory bowel disease (IBD) (22, 23).

γδ T cells are now a hot topic, and several impressive articles review the key functional roles of the γδ T cells in transplantation (24), anti-viral infection (25), and responses to the gut microbiota (26), as well as the γδ TCR in diagnosis and prognosis of hematologic tumors (27). Ribot, et al. reviewed the physiology and surveillance of γδ T cells in secondary lymphoid organs and peripheral tissues (28), and Rampoldi, et al. nicely discussed the different talks with gut microbiota of the intestinal γδ T cells in three layers, i.e., intestinal intraepithelial γδ T cells, lamina propria γδ T cells and Peyer´s patch γδ T cells (29). However, the intestinal γδ T cells may also function in the distant organs (30). This article discusses the current updates of intestinal γδ T cells in terms of their development and functions inside and outside the intestine with focus on novel functions of γδ T cells in the distant organs.

## 2 Intestinal homing of γδ T cells

Intestinal homing of γδ T cells is regulated by several key factors. In mice, the Vγ5^+^ γδ T cells express chemokine receptor CCR9 and integrin αEβ7; the CCR9 receptor binds CCL25, a chemokine highly expressed by IECs, promoting intestinal homing of Vγ5^+^ γδ T cells (31–34). CCL25 and CCR9 deficient mice show a specific decrease of γδIELs (33, 34). Integrin αE, also known as CD103, dimerizes with β7 to form a receptor complex that binds to E-cadherin expressed on IECs, promoting entry and residence of γδIELs in the intestinal epithelium (7, 35, 36). Either αE or β7 deficiency reduces γδIEL number, but a greater decrease occurs in β7 deficient mice as β7 can also dimerize with integrin α4 to form a lymphocyte homing receptor integrin α4β7 (37–40). The α4β7 receptor has two natural ligands. One is the mucosal vascular addressin cell adhesion molecule-1 (MAdCAM-1) specifically expressed on the endothelium of high endothelial venules in the gut and gut-associated lymphoid tissues (e.g., Peyer’s patches) (41, 42); the other is vascular cell adhesion molecule-1 (VCAM-1) expressed on stimulated endothelial cells of blood vessels, peripheral lymph nodes, and bone marrow (43). The CCL25 enhances the affinity of α4β7 for MAdCAM-1, but reduces the binding to VCAM-1, whereas CXCL10 works oppositely. These two chemokines distinctly regulate the active conformation of α4β7 and selective binding to MAdCAM-1 or VCAM-1 (39). The αE expression on γδIELs is regulated by the CCL25‐CCR9 axis (44, 45), as well as transforming growth factor β (TGF-β) and runt‐related transcription factor 3 (RUNX3) (46, 47).

A novel subset of γδ T cells that express both gut-homing integrins CD103 (αE) and α4β7 (CD103^+^ and α4β7^high^) has been identified in gut-draining MLNs and in intestinal epithelial and lamina propria compartments of mice with T cell-mediated colitis and spontaneous chronic intestinal inflammation (48). The CD103^+^ and α4β7^high^ γδ T cells are generated in MLNs and then mobilize to the intestine as they also express the CCR9 receptor with IEC-expressed CCL25 as a ligand (31). This subset of CD103^+^α4β7^high^ γδ T cells precede inflammation, and adoptive transfer of the CD103^+^α4β7^high^ γδ T cells dramatically enhances the accumulation of Th1 (INFγ)/Th17 (IL-17) cells in the intestine and severity of the disease. The CD103^+^α4β7^high^ γδ T cells are thus also named inflammatory γδ T cells (iγδ T). In addition, CD103^+^α4β7^high^ γδ T cells display a distinct transcriptional profile with a broad expression of cytotoxic mediators and NK cell receptors, which may endorse their inflammatory ability through induction of apoptosis and barrier dysfunction of intestinal epithelial cells (48).

## 3 Lymphopoiesis of intestinal γδ T cells

**Figure 2:**
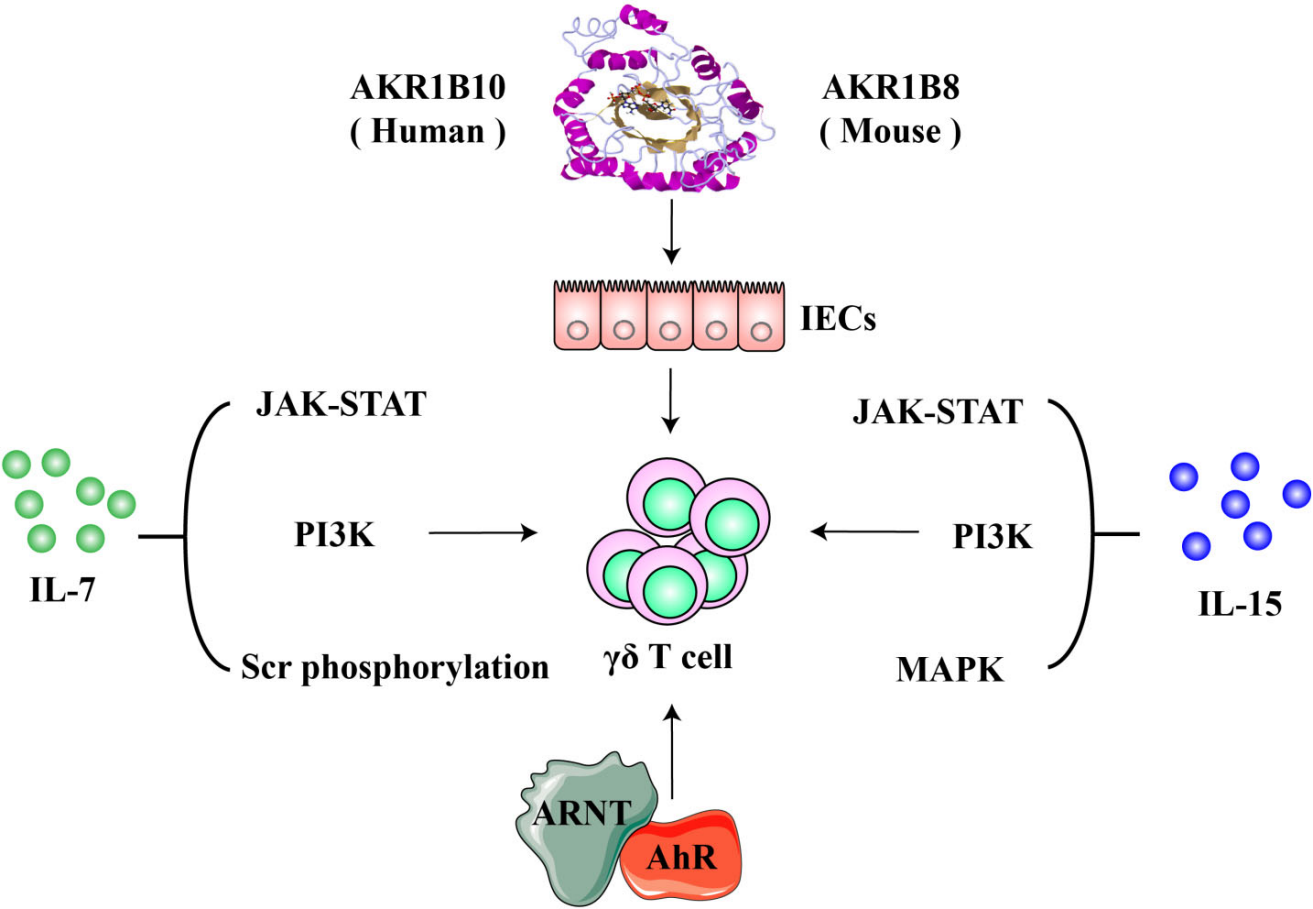
Molecules involved in the lymphopoiesis and functions of γδ T cells. IL-7 and IL-15 are critical cytokine signaling pathways that regulate the proliferation and survival, development and maturation of γδ T cells. Aryl hydrocarbon receptor (AhR) modulates γδ T cells *via* the AhR nuclear translocator (ARNT)-mediated signaling; AKR1B8 (AKR1B10 in humans) may exert regulatory effects on γδ T cells through an intestinal epithelial cell-mediated mechanisms.

Gut mucosa is a main site of extrathymic lymphopoiesis of T cells, populating mostly γδ T cells (49–52). Interaction between IECs and mucosal lymphocytes is important in the regulation of intestinal lymphopoiesis of T cells. In mice, Vγ5^+^ γδ T cells expand and transit to a mature phenotype from immature in the intestinal epithelium within the first few weeks after implanted (14). Factors that are critical for the proliferation, survival, and homeostatic maintenance of γδ T cells in the intestine include IL-7, IL-15, butyrophilin-like molecules, aryl hydrocarbon receptor, and aldo-keto reductase 1B8 (AKR1B8) (**Figure 2**). Interestingly, gut microbiota does not influence the expansion and maturation of the γδ T cells in the intestine, but stimulates their function, such as granzyme expression (53, 54).

### 3.1 Butyrophilin and butyrophilin-like molecules

After homing in the intestine, γδ T cells are shaped by butyrophilin-like (BTNL) molecules that are expressed on the surface of IECs (14, 55). By definition, butyrophilin-like proteins are similar to butyrophilin (BTN) which has two members in mice and six members in humans (56). BTNL family consists of six members in rodents and five in humans (57, 58). Several BTN/BTNL molecules are involved in immune regulation (59, 60). For instance, human BTN3A1 mediates the response of peripheral blood γδ T cells to low-molecular-mass microbial and endogenous metabolite phosphoantigens (61, 62), while SKINT1, a butyrophilin-like member expressed specifically by suprabasal keratinocytes shapes murine dendritic epidermal T cells (DETCs), i.e., Vγ3^+^ γδ T cells (63). In the gut of mice, intestinal Vγ5^+^ γδ T cells expand and mature *via* regulation of BTNL1 and BTNL6 heterocomplexes expressed on the surface of enterocytes (14). In this process, the BTNL1 and BTNL6 complex selectively promotes the phenotypic conversion of immature Vγ5^+^ γδ T cells and selective outgrowth of the mature Vγ5^+^ γδ T cells (14, 64). The BTNL1/BTNL6 complexes are expressed by post-mitotic (differentiated) enterocytes interspersed with IELs, but not by replicating epithelial progenitors, where no IELs reside. The shaping process of the intestinal Vγ5^+^ γδ T compartment seems unique by BTNL and is not affected by the thymus, lymph nodes, and Peyer’s patches, or by gut microbiota and dietary protein antigens (14). In humans, it is that the BTNL3 and BTNL8 complexes expressed by intestinal epithelial cells shape the Vδ2^−^ Vγ4^+^ γδ T cell compartment in an organ-specific manner (14), whereas BTNL2 is a negative regulator (65). Recently, it has been reported that BTNL molecules may function through direct binding to their respective γδ TCRs (66, 67). Once established in the intestine, γδ T cells rely on IEC-expressed IL-15 and dietary AhR ligands for their maintenance and survival (68–70).

### 3.2 IL-7 and IL-15 as key cytokines for lymphopoiesis and functions of γδ T cells

In the intestine, IL-7, expressed by IECs and required for γδ T cell lymphopoiesis (71, 72), signals through a heterodimer IL-7 receptor (IL-7R) composed of a unique α chain (IL-7Rα) and a common γ chain (CD132) (73–75). Binding to IL-7R, IL-7 activates JAK-STAT, PI3K, and Src phosphorylation signaling pathways to regulate target gene expression, including up-regulation of anti-apoptotic genes Bcl-2, Bcl-xL and Mcl-1 and down-regulation of pro-apoptotic genes Bax and Bak. This contributes to the survival function of IL-7, with so-called trophic effects on lymphoid progenitors and mature lymphoid cells (76–78).In mice, intestinal γδ T defects induced by IL-7 deficiency are restored by targeted expression of IL-7 in enterocytes (79). The ectopic expression of IL-7 in enterocytes does not restore γδ T cells defective in other tissues induced by the IL-7 deficiency, such as the thymus (79). IL-7R is also found in lymphocytes isolated from lamina propria and recombinant IL-7 can stimulate their growth (72). Therefore, locally expressed IL-7 plays a critical role in innate immunity against infections, such as *Citrobacter rodentium*, a mouse extracellular enteric pathogens like the human enteropathogenic *Escherichia coli* and enterohemorrhagic *Escherichia coli* (80).

IL-15 is also essential for the repertoire of intestinal γδ T cells. IL-15 signals through an IL-15 receptor (IL-15R) complex consisting of an IL-15Rα chain, an IL-2Rβ (CD122), and a γ chain. IL-15 could signal through either trans-presentation or cis-presentation. In the trans-presentation, IL-15 binds to IL-15Rα and forms an IL-15/IL-15Rα complex, which is then presented to the IL-15Rβγ complex on the membrane of neighboring cells, while IL-15 assembles a cis quaternary complex with IL-15Rα, IL-2Rβ, and γ on the cells in the cis-presentation (81, 82). The flexibility of IL-15α allows the interface of IL-15 ligand-receptor to be identical in either *cis* or *trans* (81, 83). Upon binding to its receptors, IL-15 activates JAK-STAT, PI3K, and MAPK pathways, induces expression of anti-apoptotic Bcl-2 and proto-oncogenes c-Myc, c-Fos, c-Jun, c-Myc, and NF-κB, and promotes cell proliferation and maturation (83–86). IL-15 and IL‐15Rα are expressed by enterocytes and dendritic cells in lamina propria, forming an IL-15/IL‐15Rα complex trans-presented to γδ T cells (87, 88). IECs are the main source of IL-15 in the intestine, and IEC-specific IL-15 knockout leads to a decrease in γδ T percentage and absolute number in the intestine and to impairment of functional maturation, such as the decrease in granzyme B expression, whereas IL-15 knockout in blood vascular endothelial cells (BECs) and hematopoietic cells does not affect intestinal γδ  T cells (88). IEC-specific knockout of IL-15 also leads to a decrease of γδ T cells in laminate propria, but not in the thymus. The IL-15 knockout in BECs/hematopoietic cells has no effects on γδ T cells in the thymus ether, indicating the organ specificity of the expressed IL-15 cytokine. In addition, the intestinal γδ T cells in IEC-specific IL-15 knockout mice exhibit reduced survival, but increased apoptosis due to reduced Bcl-2 but increased Fas expression (88). In mice and humans, IL-15 promotes the proliferation and cytotoxic capacity of γδ T cells, enhancing antitumor activity (89). Human Vδ2^+^ T cells isolated through TCR-crosslinking or activated by isopentenyl pyrophosphate (IPP) exhibit strong inhibition on the αβ T cell proliferation, and IL-15 can greatly enhance the inhibitory phenotype of Vδ2^+^ T cells (90). The authors believe that pharmacologic activation and expansion of Vδ2^+^ T cells through the Vδ2 TCR yields potent killer activity and suppression of αβ T cell responses as well. Makkouk, et al. recently reported that secreted IL-15 can sustain the proliferation and antitumor activity of Vδ1^+^ T cells engineered with GPC-3.CAR (91). Dendritic cell (DC) vaccine demonstrates therapeutic effects on acute myeloid leukemia, and IL-15 secreting DC cells yield more efficacy through activation of the innate cytotoxic capacity of γδ T cells (92). Together IECs act as a IL-15 niche to regulate the development, function, and homeostasis of the intestinal γδ T cells.

### 3.3 Aryl hydrocarbon receptor and dietary ligands

Aryl hydrocarbon receptor (AhR) is a ligand-activated cytosolic transcription factor receptor that uses dioxin or aromatic (aryl) hydrocarbon and endogenous indole derivative (e.g., kynurenine) as ligands. Upon binding to ligands, AhR is dissociated with chaperones, translocated into the nucleus, and dimerized with AhR nuclear translocator (ARNT) to drive the expression of target genes. The AhR signaling regulates immunity, stem cells, and cellular differentiation, involved in developmental and pathological processes (93–97). In the intestine and skin, AhR plays a crucial regulator in the survival and maintenance of γδ T cells (70). AhR deficiency or absence of AhR ligands leads to increased apoptosis and striking loss of over 95% of γδ T cells in the intestine, coupled with subsequent dysbiosis of gut microbiota and vulnerability to epithelial damage, whereas the γδ T cell subset in lymph nodes, spleen or thymus is not affected; proliferation of γδ T cells is normal, but survival is decreased in AhR deficient mice (70).

AhR contains two highly conserved, period clock-AhR nuclear transporter (Arnt)-single-minded (PAS) domains, which are primarily evolved to sense environmental changes in energy (98). Therefore, AhR activity can be regulated by dietary components, such as tryptophan-derived phytochemical I3C in cruciferous vegetables, which is converted into high-affinity AhR ligands, indolo[3,2-b] carbazole (ICZ) and 3,3-diindolylmethane (DIM) (99). Yet it is difficult to determine the exact nature of potential dietary AhR ligands due to the chemical complexity of diet, but a study has indeed shown that feeding C57BL/6 mice with a standard diet (5021-3 Autoclavable Rodent Lab Diet) significantly induces expression of the AhR target gene, *Cyp1a1*, compared to a synthetic purified diet (AIN*-*76A Purified Rodent Diet); consistently, mice fed with the synthetic diet exhibit a significant decrease in γδ T cells in the small intestine compared to mice with the standard diet. In contrast, dietary supplementation of phytochemical I3C in mice fed with the synthetic diet activates AhR and induces *Cyp1a1* expression, coupled with recovery of γδ T cells in the small intestine (70).

### 3.4 Aldo-keto reductase 1B10/aldo-keto reductase 1B8

Aldo-keto reductase 1B10 (AKR1B10) is a cytosolic protein that is specifically expressed in the intestinal epithelial cells, where it protects host cells from luminal and cellular carbonyl lesions and promotes fatty acid/lipid synthesis, regulating AKT and ERK signaling pathways (100–102). Aldo-keto reductase 1B8 (AKR1B8) is the orthologue in mice of human AKR1B10 (103). AKR1B10 expression is lost or markedly reduced in ulcerative colitis and associated colorectal cancer (104); siRNA-mediated silencing of AKR1B10 inhibits epithelial cell proliferation (105) and targeted disruption of AKR1B8 locus leads to abnormal self-renewal of the intestinal epithelium and high susceptibility to dextran sulfate sodium (DSS)-induced colitis and associated tumorigenesis (104). More importantly, AKR1B8 deficient mice in naïve status demonstrate severe abnormalities in γδ T cell development and function, accompanied by abnormal antigen presentation and effector T cell development (106).

### 3.5 Other molecules and factors

G protein-coupled receptor GPR18 regulates γδ T cell abundance in the gut and the positioning next to epithelial cells, rather than to laminate propria (107, 108). In sharp contrast, GPR55 negatively regulates intestinal γδ T cells as GPR55 deficient mice show an increase in the number and migration of γδ T cells, and their crosstalk with epithelial cells as well (109).

Intestinal flora does not have effects on the development and proliferation of γδ T cells (53), but affects their function, promoting γδ T cytotoxicity and antimicrobial function, such as the expression of regenerating islet-derived protein 3 Gamma (RegIIIγ) (110, 111). RegIIIγ (RegIIIα in humans) is an antimicrobial peptide (AMP) that inhibits Gram-positive bacteria (112, 113). The intestinal γδ T cells thus act as an early responder that restricts the intestinal bacterial penetration into mucosa after epithelial injury.

## 4 TCR ligands and activation of intestinal γδ T cells

**Figure 3:**
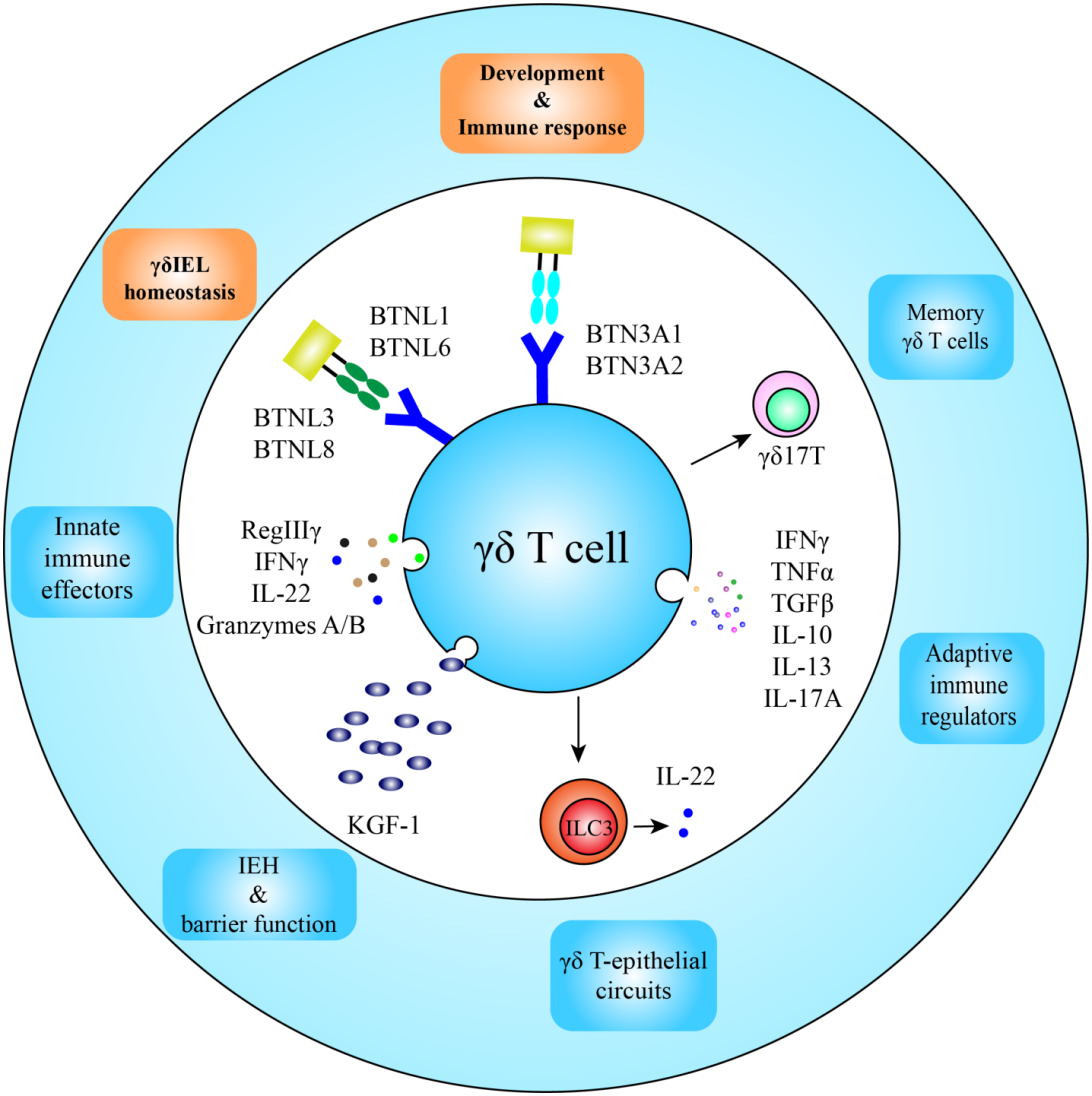
Function and development of intestinal γδ T cells. After homing in the intestine, butyrophilin and butyrophilin-like molecules participate in development and homeostatic maintenance of γδ T cells; and located in the frontline of the intestinal defense, intestinal γδ T cells are the first immune responders to appear in infection spots, functioning as innate immune effectors and adaptive immune regulators as well. Intraepithelial γδ T cells also secrete KGF-1 and IL-22 for intestinal epithelial homeostasis (IEH) and integrity of the epithelium. Some γδ T cells in the lamina propria may obtain long-lived memory phenotypes.

The γδ TCRs are the main molecules on the surface of γδ T cells involved in the recognition of antigens and pathological conditions, and the complementarity defining region 3 (CDR3) comprises the most diversity of the receptors (114). However, in striking contrast to αβ T cells which are activated in an MHC-restricted manner, the majority of γδ T cells are activated in an MHC-independent mechanism, requiring neither MHC-mediated antigen presentation, nor co-receptor interaction (114). The antigens recognized by most γδ T cells remain baffling, which may be derived from the high challenges in the identification of the γδ TCR antigens. As the lack of general restricting molecules, the antigens could be any molecules present on the cell surface or in the surrounding extracellular space, such as proteins, carbohydrates, lipids, and nucleic acids. This extremely increases the complexity of antigen identification. In addition, the affinity of γδ TCRs to their antigens is low at micromolar levels and thus classical strategies of protein biochemistry for antigen identification may not be applied (115). Alternative methods, such as blocking antibodies and genetic approaches, are tedious and labor-intensive, and usually need prior knowledge of possible candidates (115–118). Despite these hurdles, proceedings of γδ TCR antigen identification have been achieved, and γδ TCR antigens identified thus far include MHC-like molecules, such as MHC-Ib molecule T10/T22 (114, 119), lipid antigen-presenting molecules CD1-c and CD1-d, cell stress-induced Annexin A2 and ephrin receptor A2 (EphA2), and butyrophilin molecules (**Figure 3**). Please read the review article (114) for more details on γδ TCR antigens.

BTNL1 and BTNL6 heterodimers are involved in the shaping of Vγ5^+^ γδIELs in the mouse gut, whereas the BTNL3 and BTNL8 complex is involved in the development of human intestinal Vγ4^+^ γδIELs (14). Recent work has revealed the direct binding and interaction mode of BTNL proteins with their respective γδ TCRs through germline-encoded Vγ4 complementarity-determining region 2 (CDR2) and HV4 loops in variable γ-chain (66, 67). The other CDRs are not involved in BTNL protein binding, but are available for clonally specific ligand binding, such as CD1-d.

## 5 Functions of intestinal γδ T cells in the gut and distant organs

The intestinal γδ T cells have been a hot topic of intestinal immunity, and novel functions of this special type of cells, in the gut and distant organs, have been increasingly revealed.

### 5.1 Intestinal γδ T cell functions in the gut

Located in the mucosa, the intestinal γδ T cells are the first immune cells to appear in many bacterial infections and shape adaptive immune response, being a critical component of intestinal mucosal immunity (**Figure 3**). **Table 1** summarizes the main functions of the intestinal γδ T cells with references.

**Table 1:** Functions of intestinal γδ T cells in the gut and distant organs.

Locations	Functions	References
In the gut	Homeostasis and barrier function of the intestinal epithelium	(114–119)
	Surveillance of intestinal infections	(49, 104, 105, 120–138)
	Epithelial cell remodeling and responding to diets	(139, 140)
	Development and progression of colorectal cancer	(105, 114)
	Ischemic brain injury repair	(24, 141, 142)
In the distant organs	Psychosocial stress responses	(143)
	Fracture repair	(144)

#### 5.1.1 Homeostasis and barrier function of the intestinal epithelium

Intestinal γδ T cells modulate homeostasis of intestinal epithelium by expression of keratinocyte growth factor 1 (KGF-1). KGF-1, also called FGF-7, promotes proliferation, maturation, and injury repair of IECs and regulates tight junctions and mucosal permeability (120–122). In mice, Vγ5^+^ γδ T cells are necessary and sufficient for integrity maintenance of the epithelial tight junctions after enteric infection, such as *Salmonella enterica* (123). In *TCRδ -/-* (γδ T cell deficiency) and *KGF-1 -/-* mice, the proliferation and migration of intestinal epithelial cells are decreased, and the permeability of intestinal mucosa is increased (121, 123). These mice are sensitive to colitis induced by DSS with severe epithelial damage and impaired injury repair (120–122). The intestinal γδ T cells also produce IL-22 (124), which can stimulate the secretion of antimicrobial peptides (AMP) from IECs and contribute to the repair of injury (125). Therefore, the intestinal γδ T cells play an important role in homeostasis and wound repair of the intestinal mucosa.

#### 5.1.2 Surveillance of intestinal infections

Intestinal γδ T cells are dynamic and constantly migrate within epithelium *via* occludin-mediated cell–cell contact to perform surveillance of epithelium (126, 127). At steady status, γδ T cells distribute in the middle region of intestinal villi, where they reside between the basement membrane and epithelial layer, but also migrate to the intercellular space between IECs for short-time surveillance. An individual γδ T cell surveys a large area and contacts numerous IECs in a short time (54, 127). In response to the invasion of bacteria or parasites, γδ T cells gather in pathogen-rich areas rapidly and reduce normal surveillance behavior, accompanied by increase of “flossing” movements into lateral intercellular space between the IECs (54, 127, 128). The exact function of γδ T cell flossing is unknown yet, but its association with pathogen invasion suggests a crucial role in infection control and epithelial repair. MyD88 signaling in IECs is a key regulator in sensing invasive pathogens and subsequent behavioral changes of γδ T cells; specific blockage of the MyD88 signaling pathway in IECs rigorously blunts the γδ T cell response (54, 110). Gut commensals have no effects on γδ T cell number, but may contribute to their distribution within villi and to their migratory behavior and antimicrobial activity (54, 110, 111). In short, γδ T cells survey epithelial integrity, whereas IECs dictate γδ T cell behavior and facilitate adaptation in the intestinal milieu.

Microbial infection constitutes a major challenge that the intestinal epithelium encounters. Anti-infection immunity of γδ T cells includes innate and adaptive responses, and the anatomical location of intestinal γδ T cells grants them a privilege to isolate and restrict microbial pathogens from the entrance into the systemic compartment, building up the frontline of defense. Intestinal γδ T cells can express AMP (e.g., RegIIIγ) to control pathogens (110) or IL-22 to promote AMP expression by IECs (129). γδ T cells also express cytolytic factors, e.g., granzyme A and B and perforin to lyse infected or transformed intestinal cells (111, 130–132). Activated γδ T cells can also prevent potently against intracellular pathogens through an interferon-mediated mechanism, including IFNγ, type I (IFNα), and type III interferons (133, 134). Cytotoxic potentials of γδ T cells are regulated by cell surface receptors, e.g., γδTCR and NKG2D (natural killer group 2D) (135, 136), and DNAX accessory molecule-1 (DNAM-1), leukocyte function-associated antigen-1 and co-stimulatory receptor CD27 are involved in the cytotoxicity of γδ T cells (137).

In addition to the innate response, intestinal γδ T cells quickly recruited to the inflammatory sites also shape the early immune events through secretion of a variety of cytokines to promote recruitment and activation of dendritic cells, phagocytes, neutrophils, B cells, and conventional T lymphocytes (19, 138). Cytokines secreted by intestinal γδ T cells include IFNγ, TNFα, TGFβ, IL-10, IL-13, IL-17A and prothymosin β4 (7). IFNγ, TNFα, and IL-17A are important pro-inflammatory factors whereas IL-10, TGFβ, KGF-1, and prothymosin β4 are anti-inflammatory cytokines, promoting healing and integrity of intestinal epithelium (7, 17, 122, 139–141, 145). Therefore, intestinal γδ T cells play a dual role in microbial infection, i.e., inhibiting microbial invasion by induction of inflammation in the early stage but limiting excessive inflammation and tissue damage in the later stage. In the different stages of colitis, therefore, intestinal γδ T cells seem to play a different role, i.e., a pathogenic role in the early stage, but a protective role in the later stage (142, 143).

#### 5.1.3 Epithelial cell remodeling responding to diets

Enzymes and transporters required for carbohydrate digestion and absorption are induced by high-carbohydrate diets, coupled with changes in specialized enterocyte subsets (144). Carbohydrate transcriptional re-programming and epithelial cell remodeling on demand occur rapidly within 5 days of high carbohydrate feeding in mice, and intestinal γδ T cells play a crucial role in this process through suppression of IL-22 expression by type 3 innate lymphoid cells (ILC3s) (144). In response to carbohydrate diets, tissue localization, transcriptome, and behavior of γδ T cells enriched at the barrier surface of the intestine are changed. In the intestine, the intraepithelial γδ T cells are abundant and closely interact with epithelial cells whereas the lamina propria γδ T cells are a minor population of CD45^+^ lymphocytes (146). The γδ T cells in different tissue compartments respond differentially to high carbohydrate diets. The lamina propria γδ T cells increase in frequency and number in high-protein feeding, but intraepithelial γδ T cells move more rapidly. RNA-seq analysis indicates that γδ T cells in both the epithelium and lamina propria compartments demonstrate transcriptional changes, particularly in lamina propria γδ T cells that emerge with the greatest differentials of the transcriptome. Additionally, γδ T cells move to the crypt base in response to high-carbohydrate diets to influence the transcriptome and remodeling of epithelial cells through interaction with progenitors in the crypt base (144). This γδ T-mediated epithelial remodeling may represent an important mechanism of intestinal adaption to environmental changes.

#### 5.1.4 Development and progression of colorectal cancer

It is well known of the surveillance of the γδ T cells in transformed intestinal cells through the expression of cytolytic factors, but their temporal contribution to the development and progression of colorectal cancer is unclear (111). A recent study revealed that most γδ T cells resident in pre-malignant or non-tumor colon tissues host a cytotoxic signature, while the γδ T cells infiltrated in tumor tissues exhibit a pro-tumorigenic profile; the roles of γδ T cell subsets in pro- and anti-tumor activity are associated with distinct usage of the Vγδ gene of the T cell receptor in both humans and mice (147). This novel work addresses an important question on the intestinal γδ T cells as a double-edged sword in colorectal cancer.

### 5.2 Intestinal γδ T cell functions in pathological settings of distant organs

Although located in the intestine, recent studies have revealed that the intestinal γδ T cells are important regulators of pathological settings in distant organs.

#### 5.2.1 Intestinal γδ T cells and ischemic brain injury repair

A subpopulation of γδ T cells develops a long-lasting memory phenotype and adaptive responses (148). Recent studies indicated that commensals in the gut may yield an impact on the recovery of ischemic brain injury in mice through an interleukin-17 (IL-17) producing memory γδ T (named γδ17T) mediated mechanism (30). Intestinal dysbiosis induced by antibiotics leads to changes in homeostasis of intestinal γδ17T and T_reg_ cells and trafficking of these gut effector T cells to leptomeninges after stroke, thus influencing the outcome of acute brain injuries. This is a pioneer study in the special gut-brain axis. Recently, *Wang, et al.* reported that electro-acupuncture could regulate the γδ T and Treg cells in the ischemic brain and small intestine and thus exerts protective a role on ischemic stroke (149). More recently, Piepke and colleagues reported that IL-10-mediated IL-17 production is a key factor that limits stroke lesions, and may be a potential target for stroke management (150).

#### 5.2.2 Intestinal γδ T cells and psychosocial stress responses

Gut microbiota trains the intestinal immune system to facilitate the maintenance of gut homeostasis; the gut microbiota also mediates the stress-induced impairment of brain function. A recent report proposed that the intestinal γδ T cells are important mediators in the axis of gut microbiota-stress-brain function impairment (151). A specific Lactobacillus species of gut microbiota drives the differentiation and meningeal accumulation of colonic γδ17T cells and thus modulates behavioral vulnerability to chronic social stress through a mechanism mediated by the dectin-1 signaling pathway.

#### 5.2.3 Intestinal γδ T cells and fracture repair

In fracture repair, IL-17 produced locally by γδ T cells and Th17 cells drivers the inflammatory phase. *Dar, et al.* reported recently that the gut microbiota-mediated expansion and migration to the callus of intestinal γδ T and Th17 cells are involved in fracture repair (152). The S1P-receptor-1 (S1PR1) signaling pathway regulates egress and homing to the callus of the Th17 cells; and deletion of the γδ T cells and microbiome (by antibiotics) and blockade of Th17 cell influx into the callus impair the fracture repair, suggesting the importance of γδ T and Th17 cells activation and trafficking in fracture repair. It is recently understood that IL-17A secreted by γδ T cells stimulate the proliferation of mesenchymal progenitor cells and differentiation of osteoblasts to accelerate bone formation and fracture healing; in IL-17A deficient mice, the bone fracture repair is impaired (153).

## 6 Concluding remarks

The intestinal mucosa of mammals has evolved an amazing immune compartment to protect the host from pathogenic attacks. The intestinal γδ T cells represent a specific population of cells that function in the maintenance of epithelial homeostasis, barrier integrity, damage repair, and rapid compartmentalization of microbial pathogens. In response to infections, intestinal γδ T cells function as innate immune cells to restrict microbial pathogens from systemic spreading and then shape early adaptive immune responses through the recruitment of neutrophils and activation of phagocytes and dendritic cells. Therefore, intestinal γδ T cells are key regulators of mucosal physiology and pathology in disease settings. The intestinal γδ T cells may also deliver effects on distant organs, such as brain stroke and fracture repair and psychosocial stress responses.

A mysterious question is that γδ T cells are activated independently of MHC and thus antigens of γδ TCR, as lack of restricting molecules, could be different types of molecules on the cell surface or in surrounding extracellular space. Gut microbiota would be an exciting area for the exploration of microbially originated antigens/ligands. IELs regulate both the intestinal immunity and microbiota and are thus located at top of the hierarchy that guards intestinal health. How IELs regulate homed γδ T cells would then be another interesting topic. Yet some epithelial-originated molecules, such as IL-7, IL-15, BTNL, and AhR have been characterized, but identification of AKR1B10 as a novel molecule that mediates the development and function of intestinal γδ T cells implies a warranty of further investigation. New technologies developed to date, such as single-cell RNA-seq (154) and RNAscope^®^ *in situ* hybridization combined with immunohistochemistry (155), would add avenues to understand γδ T cells in the frontline of intestinal immunity. The gut microbiota, circadian rhythms, sex hormones, and neurotransmitters are all regulatory factors of intestinal γδ T cells (156); it is challenging but intriguing to boost the beneficial and protective roles of γδ T cells but tame their proinflammatory action.

## Author contributions

G-QL researched data and wrote the draft. JX performed the revisions. WZ drew figures. WL and LL contributed to the revisions of figures. XZ and DC substantially contributed to the discussion of content and reviewed/edited/finalized the manuscript. All authors contributed to the article and approved the submitted version.
